# Role of Cytoreductive Nephrectomy in the Immune Checkpoint Inhibitor Era: A Multicenter Collaborative Study

**DOI:** 10.1111/iju.70207

**Published:** 2025-08-19

**Authors:** Takuhisa Nukaya, Kiyoshi Takahara, Shingo Toyoda, Lan Inoki, Wataru Fukuokaya, Keiichiro Mori, Takehiro Iwata, Kensuke Bekku, Ryoichi Maenosono, Takuya Tsujino, Yosuke Hirasawa, Takafumi Yanagisawa, Takeshi Hashimoto, Kazumasa Komura, Motoo Araki, Kazutoshi Fujita, Yoshio Ohno, Ryoichi Shiroki

**Affiliations:** ^1^ Department of Urology Fujita‐Health University School of Medicine Toyoake Japan; ^2^ Department of Urology Kindai University Faculty of Medicine Osaka Japan; ^3^ Department of Urology The Jikei University School of Medicine Tokyo Japan; ^4^ Department of Urology Okayama University Graduate School of Medicine Okayama Japan; ^5^ Department of Urology Osaka Medical and Pharmaceutical University Osaka Japan; ^6^ Department of Urology Tokyo Medical University Tokyo Japan

**Keywords:** cytoreductive nephrectomy, IMDC classification, immune checkpoint inhibitor, neutrophil‐to‐lymphocyte ratio, sarcomatoid differentiation

## Abstract

**Objectives:**

We aimed to evaluate overall survival (OS) and determine the optimal timing of cytoreductive nephrectomy (CN) in patients with metastatic renal cell carcinoma (mRCC) receiving immune checkpoint inhibitor (ICI)‐based therapy.

**Methods:**

This retrospective study reviewed medical records of 447 patients with mRCC treated with ICI at multiple Japanese institutions between January 2018 and August 2023. From this cohort, 178 patients with lymph node or distant metastases received either cytoreductive nephrectomy (CN group; *n* = 72) or ICI therapy without cytoreductive nephrectomy (non‐CN group; *n* = 106) as first‐line treatment.

**Results:**

Median progression‐free survival was 15.7 months, and median overall survival was 58.1 months. CN significantly improved OS, with the CN group's median OS not reached, compared to 29.6 months in the non‐CN group (*p* = 0.01). Deferred CN also showed improved survival outcomes. Poor prognostic factors for immediate CN included International Metastatic Renal Cell Carcinoma Database Consortium poor risk, sarcomatoid differentiation, and a high neutrophil‐to‐lymphocyte ratio.

**Conclusions:**

We developed a prognostic model to guide patient selection for CN, emphasizing the need for personalized treatment strategies.

**Abbreviations**
CI: confidence interval
CN: cytoreductive nephrectomy
CT: computed tomography
ICI: immune checkpoint inhibitor
IMDC: International Metastatic Renal Cell Carcinoma Database Consortium
INF: interferon
IQR: interquartile range
KPS: Karnofsky Performance Status
mRCC: metastatic renal cell carcinoma
NLR: neutrophil/lymphocyte ratio
OS: overall survival
PFS: progression free survival
TKI: tyrosine kinase inhibitor

## 1 Introduction

In 2015, nivolumab became the first immune checkpoint inhibitor (ICI) approved for the treatment of metastatic renal cell carcinoma (mRCC) [1]. Following this milestone, combination therapies involving ICI therapy, such as ICI‐ICI and ICI + tyrosine kinase inhibitors (ICI‐TKI), were subsequently introduced [2, 3, 4], marking the beginning of the ICI era and dramatically transforming the treatment landscape of mRCC. However, the clinical significance of cytoreductive nephrectomy (CN), which involves the removal of the primary tumor, remains unclear in this new era.

During interferon and TKI eras, CN was demonstrated to improve survival outcomes [5, 6, 7]. However, the phase III randomized CARMENA trial challenged this understanding by demonstrating that sunitinib monotherapy was noninferior to CN combined with sunitinib [8]. In contrast, the validity of the CARMENA trial in the ICI era has been called into question, and emerging data on the combination of CN and ICI therapy have recently been reported. These studies increasingly suggest the efficacy of CN in the ICI era [9, 10, 11]. Given these developments, clarifying the role and optimal timing of CN in the ICI era, particularly in the context of clinical practice in Japan, is crucial. Currently, drug therapy for mRCC predominantly revolves around ICI therapy. However, comprehensive studies examining the role of CN in the ICI era remain limited, underscoring the need for further investigation. To address this gap, we aimed to evaluate overall survival (OS) according to the timing of CN in patients with mRCC treated in the era of ICI therapy.

## 2 Methods

We retrospectively reviewed the medical records of 447 patients with mRCC who were treated with ICI therapy at multiple Japanese institutions, including the Jikei University School of Medicine (Tokyo, Japan), Okayama University (Okayama, Japan), Tokyo Medical University (Tokyo, Japan), Osaka Medical and Pharmaceutical University (Osaka, Japan), Kindai University Faculty of Medicine (Osaka, Japan), Fujita Health University School of Medicine (Aichi, Japan), and 15 affiliated hospitals. The study period spanned from January 2014 to August 2023. From this cohort, we included 178 patients who presented with lymph node metastasis or distant metastasis at the time of initial diagnosis and received either CN or ICI therapy as first‐line treatment. Among these, 72 patients were treated with CN, while 106 received ICI therapy without CN. Treatment efficacy was assessed according to RECIST criteria, using computed tomography (CT) scans, general physical condition, and laboratory findings. To minimize selection bias between the CN and non‐CN groups, propensity score matching was performed. Propensity scores were estimated using a logistic regression model that included International Metastatic Renal Cell Carcinoma Database Consortium (IMDC) risk factors and histological subtype as covariates. One‐to‐one nearest‐neighbor matching without replacement was conducted using a caliper width of 0.2 standard deviations of the logit of the propensity score. This study was conducted in accordance with the ethical principles outlined in the Declaration of Helsinki for research involving human subjects and was approved by the institutional review board of the principal institution (Osaka Medical and Pharmaceutical University, Osaka, Japan; approval number: RINS750‐2571).

### 2.1 Statistical Analysis

Differences in variable distributions among groups were analyzed using the chi‐squared (*χ* ^2^) test for categorical variables and the Mann–Whitney *U* test for continuous variables. OS was defined as the duration from the initiation of first‐line treatment to death from any cause. Progression‐free survival (PFS) was defined as the duration from the initiation of first‐line treatment to either disease progression or death from any cause, whichever occurred first. Patients who had not experienced the terminal event were censored at the date of their last follow‐up. OS and PFS were estimated using the Kaplan–Meier method, with two‐sided 95% confidence intervals (CIs) provided for point estimates. Survival curves were generated using EZR (Saitama Medical Center, Jichi Medical University, Saitama, Japan), a graphical user interface for R (The R Foundation for Statistical Computing, Vienna, Austria) [12], and compared using the log‐rank test. When a significant difference was observed among the three groups, pairwise comparisons were performed with Bonferroni correction. To identify independent prognostic factors for OS and PFS, univariate and multivariate analyses were conducted using Cox proportional hazards regression models. A *p*‐value < 0.05 was considered statistically significant. All statistical analyses were performed using EZR.

## 3 Results

### 3.1 Patient Characteristics

A total of 178 patients with mRCC, presenting with lymph node or distant metastases at initial diagnosis, were included in this analysis. The median age was 68.5 years (IQR: 59–74), with a median follow‐up duration of 15 months (IQR: 6–27). The male‐to‐female ratio was 128:50. Clinical T‐classification at the time of diagnosis was distributed as follows: T1 in 36 patients (20.2%), T2 in 31 patients (17.4%), T3 in 80 patients (45%), and T4 in 31 patients (17.4%). According to the IMDC risk classification, 106 patients (59.5%) were categorized as intermediate risk, and 72 (40.5%) as poor risk. Lymph node metastases were present in 77 patients (43.2%), and distant metastases were observed in 158 (88.7%). Sites of metastases included the lung in 116 patients (72.9%), bone in 53 (33.3%), liver in 22 (13.8%), brain in 12 (7.5%), and other sites in 41 (25.7%). Some patients presented with multiple metastatic sites. All patients received systemic chemotherapy with either ICI‐ICI or ICI‐TKI therapy. ICI‐ICI therapy was selected as the first‐line treatment in 99 patients (55%). Among ICI‐TKI therapy, pembrolizumab/axitinib was the most frequently used, accounting for 33 patients (40.7%). Pathological examination revealed that clear cell carcinoma was the predominant histological type, observed in 136 patients (76.4%), while sarcomatoid differentiation was identified in 18 patients (10.1%) (Table 1).

**TABLE 1:** Patient characteristics.

		*n* = 178
Follow up, months (IQR)		15 (6–27)
Median age, years (IQR)		68.5 (59–74)
Sex (male:female)		128:50
T stage	cT1	36
	cT2	31
	cT3	80
	cT4	31
N stage	N0	101
	N1	77
M stage	M0	20
	M1	158
CN	Yes	72
	No	106
1st line treatment	ICI‐ICI	99
	ICI‐TKI	79
IMDC	Intermediate	106
	Poor	72
Histological subtype	Clear cell	136
	Non‐clear cell	39
Sarcomatoid differentiation	Yes	18
	No	154

Abbreviations: CN, cytoreductive nephrectomy; IMDC, International Metastatic RCC Database Consortium; IQR, interquartile range.

### 3.2 Oncological Outcomes

**FIGURE 1:**
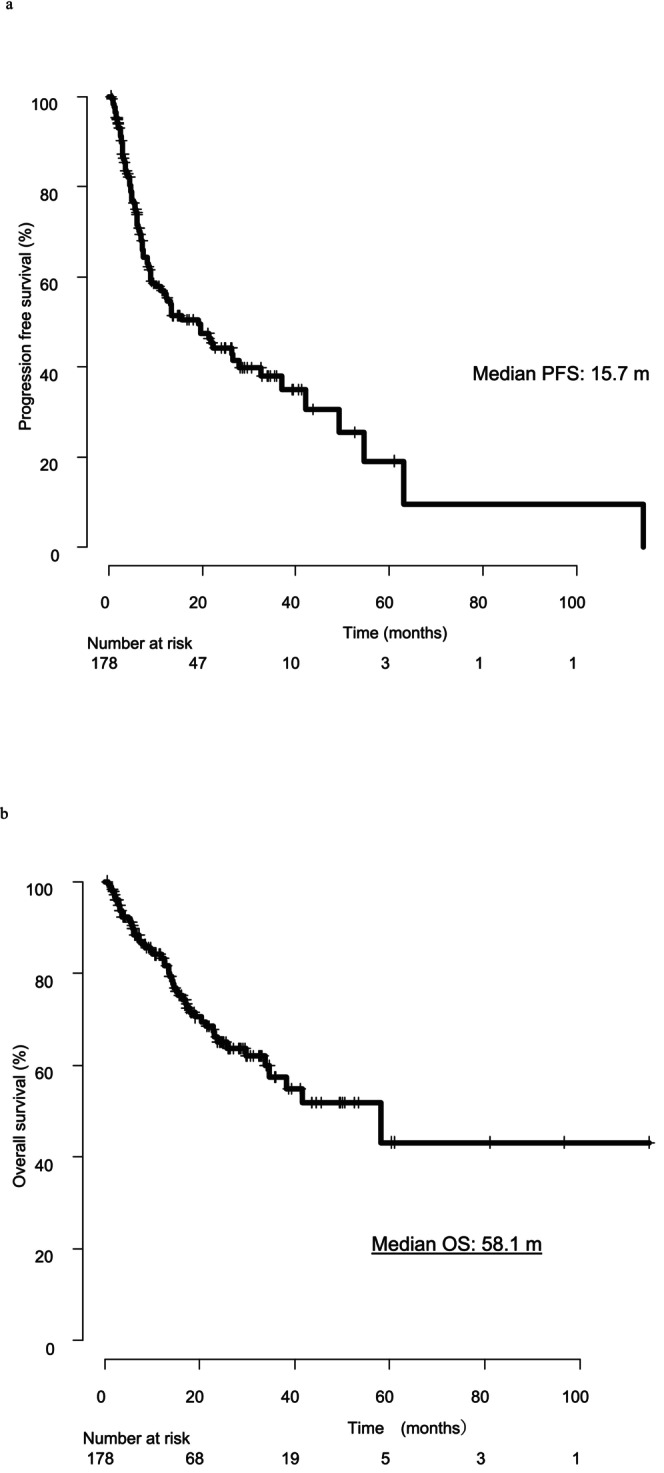
(a) Progression‐free survival (PFS) in all cases. Median PFS: 19.2 months. (b) Overall survival (OS) in all cases. Median OS: 58.1 months. OS, overall survival; PFS, progression free survival.

**FIGURE 2:**
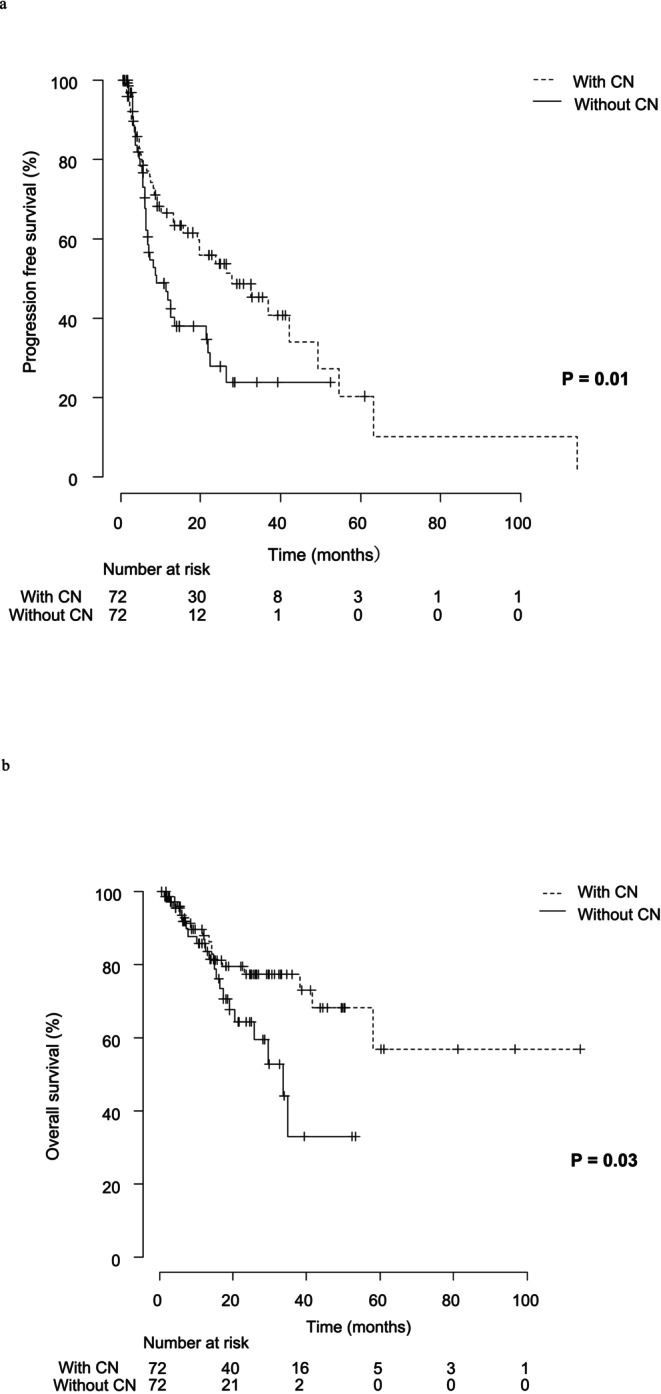
(a) Progression‐free survival in patients with and without CN. A significant difference in PFS was observed between the CN and non‐CN groups (*p* = 0.01). (b) Overall survival in patients with and without CN. A significant difference in OS was observed between the CN and non‐CN groups (*p* = 0.03). CN, cytoreductive nephrectomy; OS, overall survival; PFS, progression free survival.

**FIGURE 3:**
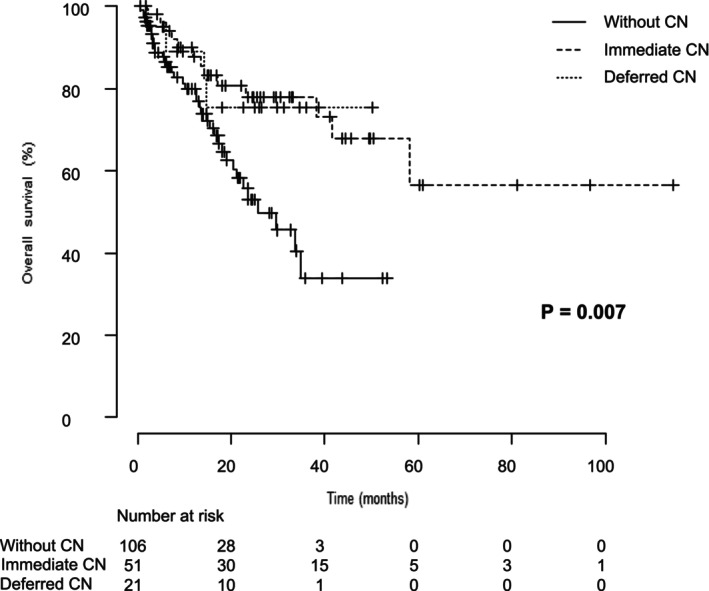
Overall survival in patients who underwent immediate CN, deferred CN, or no CN. A comparison of OS among the immediate CN group, the deferred CN group, and the without CN revealed no significant difference between the immediate CN group and the deferred CN group. However, a statistically significant difference in OS was observed when the immediate CN group was compared to the group that was without CN. (*p* = 0.007). CN, cytoreductive nephrectomy; OS, overall survival.

The median PFS for the entire cohort was 15.7 months, while the median OS was 58.1 months. Factors contributing to extended OS were analyzed across all 178 cases (Figure 1a,b). Based on univariate and multivariate analyses, four factors influencing OS were identified (Table 2). Focusing on the role of CN, we analyzed two patient groups from the cohort: the non‐CN group, consisting of 106 patients (60%) who received systemic therapy alone, and the CN group, which included 72 patients (40%) who underwent CN. The CN group was further divided into two subgroups: immediate CN (51 patients, 28.3%) and deferred CN (21 patients, 11.7%). The median time from immediate CN to the initiation of first‐line therapy was 1 month, whereas in the deferred CN group, the median time from initiation of first‐line therapy to CN was 6 months. The non‐CN group had a higher proportion of older patients, poor‐risk cases according to the IMDC classification, and patients with lymph node metastases compared to the CN group. To balance baseline characteristics, propensity score matching was performed, adjusting for IMDC classification and histological subtype (Table 3). Even after matching, the CN group demonstrated a significant improvement in PFS and OS compared to the non‐CN group. The median OS in the CN group was not reached, while in the non‐CN group, it was 29.6 months (*p* = 0.03). Additionally, the median PFS was 27.8 months in the CN group and 11.2 months in the non‐CN group (*p* = 0.01) (Figure 2a,b). Subgroup analysis according to histological subtype revealed that in patients with clear cell RCC (*n* = 136), CN was associated with a significantly longer OS (*p* = 0.03). In contrast, among patients with non‐clear cell RCC (*n* = 39), no significant difference in OS was observed between those who underwent CN and those who did not (*p* = 0.26) (Appendix S1). Further analysis compared patient characteristics among the three groups (Table 4). Although no statistically significant difference in OS was observed between the immediate CN and deferred CN groups, a significant difference was found between the immediate CN group and the non‐CN group (*p* = 0.007). The median OS for both the immediate CN and deferred CN groups was not reached, whereas for the non‐CN group, the median OS was 29.6 months (Figure 3) In this study, only patients who survived until CN were included in the deferred CN group, which may introduce immortal time bias. To minimize this bias, we performed an additional landmark analysis using the median time from initiation of first‐line therapy to surgery (6 months) as the landmark point. The results of this analysis are presented in Appendix S2. Even in the landmark analysis, the deferred CN group demonstrated a longer OS compared to the non‐CN group, supporting the findings of the primary analysis. Additionally, when comparing OS between patients treated with ICI‐ICI and ICI‐TKI therapy as first‐line treatment following immediate or deferred CN, no statistically significant difference was observed between the two groups (Appendix S3). In this mRCC cohort, the objective response rate (ORR) was 59.3% in the non‐CN group, 60.0% in the immediate‐CN group, and 61.9% in the deferred‐CN group, with no statistically significant differences observed among the three groups.

**TABLE 2:** Uni‐ and multivariate Cox proportional hazards regression models for overall survival.

	Univariate		Multivariate	
	HR (95% CI)	*p*	HR (95% CI)	*p*
Female (yes)	1.22 (0.65–2.26)	0.53		
Age ≥ 65 (yes)	1.1 (0.62–1.95)	0.73		
IMDC poor (yes)	2.41 (1.38–4.19)	0.0017	1.77 (0.99–3.17)	0.05
Metastasis (yes)	1.06 (0.48–2.37)	0.87		
N positive (yes)	1.83 (1.06–3.17)	0.029	1.69 (0.96–2.94)	0.05
T3 over (yes)	0.91 (0.51–1.57)	0.72		
KPS ≤ 70 (yes)	2.14 (1.13–4.02)	0.018	1.92 (0.97–3.81)	0.05
ICI‐ICI (yes)	1.95 (1.05–3.6)	0.033	1.44 (0.75–2.73)	0.26
CN (yes)	0.39 (0.21–0.71)	0.0023	0.42 (0.22–0.79)	0.007

Abbreviations: CN, cytoreductive nephrectomy; IMDC, International Metastatic RCC Database Consortium; KPS, Karnofsky Performance Status.

**TABLE 3:** Patient characteristics stratified by the presence or absence of cytoreductive nephrectomy (CN).

		ALL			PS‐match		
		With CN *n* = 72	Without CN *n* = 106	*p*	With CN *n* = 72	Without CN *n* = 72	*p*
Age median, (IQR)		68 (56.75–73)	69.5 (61–74)	0.09	68 (56.75–73)	70 (61.75–75.25)	0.09
Sex	Male	54	74	0.49	54	48	0.36
	Female	18	32		18	24	
T classification	cT1	17	19	0.45	17	11	0.51
	cT2	14	17		14	14	
	cT3	32	48		32	33	
	cT4	9	22		9	14	
N classification	N0	47	54	0.065	47	40	0.31
	N1	25	52		25	32	
M classification	M0	11	9	0.22	11	5	0.19
	M1	61	97		61	67	
IMDC	Intermediate	49	57	0.063	49	49	1
	Poor	23	49		23	23	
Histological subtype	Clear	62	74	0.028	62	62	1
	Non‐clear	10	29		10	10	
1st‐line treatment	ICI‐ICI	40	59	1	40	37	0.74
	ICI‐TKI	32	40		32	35	
KPS	KPS > 80	59	88	0.84	59	62	0.65
	KPS ≤ 70	13	18		13	10	

Abbreviations: CN, cytoreductive nephrectomy; IMDC, International Metastatic RCC Database Consortium; IQR, interquartile range; PS‐match, propensity score matching.

**TABLE 4:** Patient characteristics among the immediate cytoreductive nephrectomy (CN), deferred CN, and without CN groups.

		Immediate CN *n* = 51	Deferred CN *n* = 21	Without CN *n* = 106	*p*
Median age, years (IQR)		68 (54.5–73)	68 (61–71)	69.5 (61–74)	0.12
Sex	Male	38	16	74	0.78
	Female	13	5	32	
T stage	cT1	15	2	19	0.35
	cT2	9	5	17	
	cT3	22	10	48	
	cT4	5	4	22	
N stage	N0	31	16	54	0.085
	N1	20	5	52	
M stage	M0	8	3	9	0.31
	M1	43	18	97	
1st‐line treatment	ICI‐ICI	29	11	59	0.95
	ICI‐TKI	22	10	47	
IMDC	Intermediate	38	11	57	0.03
	Poor	13	10	49	
Histological subtype	Clear	43	19	74	0.08
	Non‐clear	8	2	29	
KPS	KPS ≥ 80	41	18	88	0.84
	KPS ≤ 70	10	3	18	

Abbreviations: CN, cytoreductive nephrectomy; ICI, Immune Checkpoint Inhibitors; IMDC, International Metastatic RCC Database Consortium; IQR, interquartile range; TKI, tyrosine kinase inhibitors.

### 3.3 Poor Prognostic Factors for CN

**FIGURE 4:**
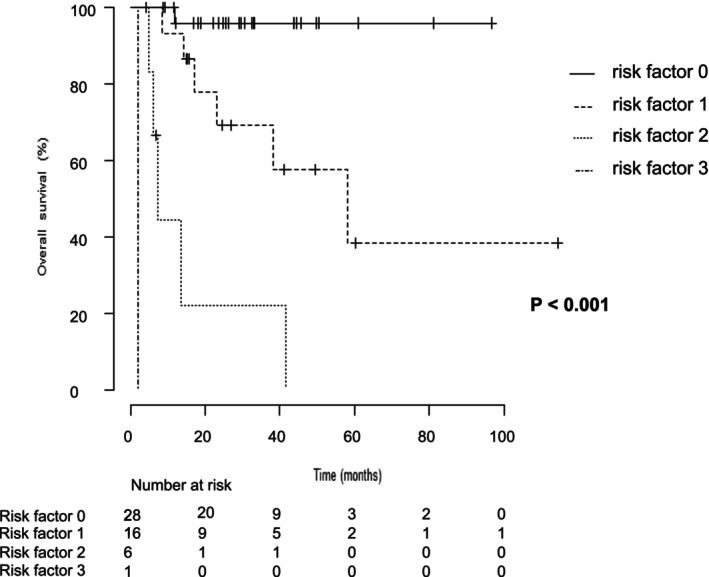
Overall survival in patients who underwent immediate CN, stratified by risk factors. In the group that underwent immediate CN, IMDC poor risk, sarcomatoid differentiation, and NLA ≥ 4 were identified as poor prognostic factors for OS. A comparison of OS between patients with 0–1 risk factor and those with 2–3 risk factors revealed a statistically significant reduction in OS for the group with 2–3 risk factors (*p* < 0.001).CN, cytoreductive nephrectomy; IMDC, International Metastatic RCC Database Consortium; NLA, neutrophil‐to‐lymphocyte ratio; OS, overall survival.

In the current study, no specific factors were identified that contributed to OS extension in patients undergoing immediate CN. However, based on the results of univariate and multivariable analyses, IMDC poor risk, sarcomatoid differentiation, and a neutrophil‐to‐lymphocyte ratio (NLR) ≥ 4 were identified as poor prognostic factors in this subgroup (Table 5). When these three factors were analyzed as risk factors, the OS was not reached for patients without any risk factor. For patients with one risk factor, the median OS was 58.1 months. In contrast, for those with two or three risk factors, the median OS was significantly shortened to 7.1 and 2.1 months, respectively. These findings suggest that in cases where two or more risk factors are present, immediate CN results in a marked reduction in OS (Figure 4). Therefore, the decision to perform immediate CN should be reconsidered on a case‐by‐case basis. Lastly, in this study, the deferred CN group demonstrated prolonged OS compared to the non‐CN group. However, factors contributing to OS prolongation or poor prognostic factors in this group could not be identified (Appendix S4).

**TABLE 5:** Uni‐ and multivariate Cox proportional hazards regression models for overall survival in the immediate cytoreductive nephrectomy (CN) group.

	Univariate		Multivariate	
	HR (95% CI)	*p*	HR (95% CI)	*p*
Female (yes)	0.67 (0.14–3.04)	0.61		
Age ≥ 65 (yes)	1.01 (0.32–3.1)	0.98		
IMDC poor (yes)	9.37 (2.79–31.4)	0.0002	17.27 (2.65–112)	0.002
Liver metastasis (yes)	3.14 (0.84–11.6)	0.087		
N positive (yes)	2.54 (0.83–7.8)	0.11		
KPS ≤ 70 (yes)	3.74 (1.17–11.9)	0.025	0.48 (0.08–2.91)	0.43
Alb ≤ 4.0 mg/dL (yes)	2.46 (0.78–7.7)	0.12		
LDH ≥ 220 IU/L (yes)	1.93 (0.63–5.94)	0.25		
Clear‐cell carcinoma (yes)	0.51 (0.13–1.89)	0.31		
Sarcomatoid change (yes)	7.81 (2.13–28.6)	0.0019	44.3 (5.53–354)	0.0003
NLR ≥ 4.0 (yes)	3.73 (1.22–11.36)	0.02	7.38 (1.31–41.37)	0.02
CRP ≥ 4.0 mg/dL (yes)	4.03 (1.01–16.05)	0.04	1.99 (0.34–11.53)	0.43

Abbreviations: Alb, albumin; CI, confidence interval; CRP, C‐reactive protein; HR, hazard ratio; IMDC, International Metastatic RCC Database Consortium; KPS, Karnofsky Performance Status; LDH, lactate dehydrogenase; NLR, neutrophil‐to‐lymphocyte ratio.

## 4 Discussion

We evaluated the utility of CN in patients with mRCC treated with therapy including ICI. Our analysis demonstrated that, regardless of whether CN was performed as immediate or deferred CN, OS was significantly prolonged compared to patients who did not undergo CN. In contrast, the ORR to first‐line therapy was similar among the immediate CN, deferred CN, and without CN groups, suggesting that CN itself did not contribute to tumor shrinkage during initial treatment. The observed improvement in OS is likely attributable to factors other than tumor response, such as patient background, general condition, or changes in the immune microenvironment [13].

In the immediate CN group, three factors—sarcomatoid differentiation, IMDC poor risk, and NLR ≥ 4 were—strongly associated with poor prognosis. Patients with two or more of these factors tended to have shorter OS than those without CN group. These findings indicate that immediate CN is not beneficial for all patients with mRCC, highlighting the need for careful patient selection when considering this procedure. Recent studies have also reported several adverse prognostic factors for immediate CN, including a Karnofsky Performance Status of ≤ 70, elevated lactate dehydrogenase levels, metastases to multiple organs, bone, liver, or brain metastases, and non‐clear cell histology [10, 14, 15, 16]. In addition, the SCREEN score, which uses hematological and oncological parameters to assess the suitability of immediate CN, has been proposed and is attracting attention for its usefulness in risk stratification. The SCREEN score consists of the following seven parameters and stratifies prognosis based on the presence or absence of each factor. The parameters are as follows: (1) the presence of three or more metastatic sites, (2) a maximum diameter of metastasis ≥ 5 cm, (3) the presence of bone metastasis, (4) the presence of systemic symptoms (such as fever or weight loss), (5) anemia, (6) hypoalbuminemia, and (7) a NLR ≥ 4 [17]. Patients who meet four or more of these criteria have been reported to experience a markedly increased postoperative mortality rate following immediate CN, making the SCREEN score an extremely important risk model for determining surgical eligibility.

In our study also, sarcomatoid differentiation and elevated NLR were significantly associated with poor prognosis following immediate CN. The introduction of comprehensive assessment tools such as the SCREEN score may serve as a valuable aid for clinical decision‐making regarding the indication for CN in the future.

With respect to deferred CN, recent studies have suggested that it may provide a greater OS benefit compared to immediate CN [18, 19, 20, 21]. The advantages of deferred CN include the ability to assess response to initial therapy before determining surgical eligibility, as well as the opportunity to administer preoperative treatment according to plan in all patients. However, due to selection of patients with favorable responses or good general condition for CN, patient selection bias is unavoidable, and careful interpretation of the results is required. In the present study, to minimize the impact of this bias, we conducted a landmark analysis excluding patients who died within 6 months of initiating first‐line therapy. Nevertheless, no significant difference in OS was observed between the immediate CN and deferred CN groups. The lack of identified poor prognostic factors in the deferred CN group may be attributable to the structural exclusion of more severe cases. In addition, immunological changes induced by CN may also contribute to the extension of survival. It has been hypothesized that removal of the primary tumor reduces the burden of tumor antigens and suppresses the secretion of immunosuppressive factors, thereby enhancing the efficacy of ICI therapy [13, 22, 23]. However, in environments characterized by persistent excessive inflammation, such as in patients with elevated NLR, the immune‐enhancing effects of CN may be offset, potentially leading to reduced efficacy of ICI therapy. Given these considerations, determining the indication for CN based on a comprehensive assessment—including hematologic biomarkers—is expected to become increasingly important in the future [24, 25, 26].

The greatest clinical significance of this study lies in its clarification of the characteristics of patients for whom immediate CN should be avoided. In particular, our findings suggest that immediate CN may not only be beneficial but also could be detrimental in patients with mRCC having features such as sarcomatoid differentiation, IMDC poor risk, or elevated NLR. In contrast, evidence regarding the selection criteria for eligible patients and the optimal timing of deferred CN remains insufficient; further validation through prospective studies is warranted. The ongoing SEVURO‐CN trial is expected to provide important insights into the optimization of both the timing and indications for CN [27].

This study has some limitations. First, the analysis was based on a relatively small cohort, and as shown in Figures 2, 3, 4, the number of cases in the subgroup analyses was limited. Therefore, the validity of the predictive models and the generalizability of the results are constrained. Second, this study was conducted primarily at tertiary care centers such as university hospitals, and patient backgrounds may differ from those seen in community healthcare settings. Additionally, the deferred CN group included both patients for whom CN was planned from the outset and those whose treatment strategy was changed after a favorable response to initial therapy. This heterogeneity may have influenced the outcomes and should be taken into consideration. In the future, larger and prospective cohort studies are needed to optimize the indications, timing, and prognostic factors for CN in patients with mRCC.

In conclusion, this study demonstrated a prognostic model for patients with mRCC undergoing immediate CN. This model enables stratification of patients into three subgroups with distinct prognoses and provides valuable information to guide decision‐making regarding immediate CN.

## Author Contributions

**Takuhisa Nukaya:** conceptualization, methodology, data curation, investigation, writing – original draft, visualization, project administration, formal analysis. **Kiyoshi Takahara:** supervision, formal analysis, writing – review and editing. **Shingo Toyoda:** writing – review and editing, supervision. **Lan Inoki:** supervision, writing – review and editing. **Wataru Fukuokaya:** supervision, writing – review and editing. **Keiichiro Mori:** supervision, writing – review and editing. **Takehiro Iwata:** supervision, writing – review and editing. **Kensuke Bekku:** supervision, writing – review and editing. **Ryoichi Maenosono:** supervision, writing – review and editing. **Takuya Tsujino:** supervision, writing – review and editing. **Yosuke Hirasawa:** supervision, writing – review and editing. **Takafumi Yanagisawa:** supervision, writing – review and editing. **Takeshi Hashimoto:** supervision, writing – review and editing. **Kazumasa Komura:** supervision, writing – review and editing. **Motoo Araki:** supervision, writing – review and editing. **Kazutoshi Fujita:** supervision, writing – review and editing. **Yoshio Ohno:** supervision, writing – review and editing. **Ryoichi Shiroki:** supervision, writing – review and editing.

## Disclosure

The authors have nothing to report.

## Ethics Statement

This retrospective chart review study involving human participants was conducted in accordance with the ethical standards of the institutional and national research committees, as well as the 1964 Helsinki Declaration and its later amendments or comparable ethical standards. The Human Investigation Committee (IRB) of Osaka Medical and Pharmaceutical University approved this study.

## Consent

Written informed consent was obtained from the parents.

## Conflicts of Interest

Kazutoshi Fujita has received honoraria from Ono, Bristol‐Myers Squibb, MSD, Eisai, Pfizer, Merck, and Takeda. Ryoichi Shiroki, Motoo Araki, Kazutoshi Fujita, Yoshio Ohno, and Kiyoshi Takahara are Editorial Board members of the International Journal of Urology and also coauthors of this article. To minimize potential bias, all of them were excluded from the editorial decision‐making process related to the acceptance of this article for publication.

## Supporting information


**Appendix S1:** (a) Overall survival by CN status in clear cell RCC. (b) Overall survival by CN status in non‐clear cell RCC.

**Appendix S2:** Landmark analysis of overall survival.

**Appendix S3:** (a) OS of first‐line treatment following immediate CN (IO+IO or IO+TKI). (b) OS of first‐line treatment following deferred CN (IO‐IO or IO‐TKI).

**Appendix S4:** Uni‐ and multivariate Cox proportional hazards regression models for OS in the deferred CN group.
