# Supplementary material for: Role of Cytoreductive Nephrectomy in the Immune Checkpoint Inhibitor Era: A Multicenter Collaborative Study
Source: Int J Urol. 2025 Aug 19;32(11):1677–85. doi: 10.1111/iju.70207 (PMC12586762; doi:10.1111/iju.70207)
Supplement: Supplementary file 1 — Appendix S1: (a) Overall survival by CN status in clear cell RCC. (b) Overall survival by CN status in non‐clear cell RCC. [file IJU-32-1677-s003.docx]

Online Resource 1a

Overall survival by CN status in clear cell RCC

0

20

40

60

80

100

0

20

40

60

80

100

Time (months)

Overall survival (%)

62

35

14

5

3

1

74

20

2

0

0

0

With CN

Without CN

Number at risk

With CN

Without CN

**ｐ=0.03**

CN: Cytoreductive nephrectomy

**ｐ=0.03**

RCC: Renal cell carcinoma

Online Resource 1b

Overall survival by CN status in non-clear cell RCC

0

10

20

30

40

50

0

20

40

60

80

100

10

6

5

3

2

1

29

11

8

3

1

0

With CN

Without CN

Number at risk

Overall survival (%)

Time (months)

With CN

Without CN

**ｐ=0.26**

CN: Cytoreductive nephrectomy

**ｐ=0.03**

RCC: Renal cell carcinoma
