# Supplementary material for: Role of Cytoreductive Nephrectomy in the Immune Checkpoint Inhibitor Era: A Multicenter Collaborative Study
Source: Int J Urol. 2025 Aug 19;32(11):1677–85. doi: 10.1111/iju.70207 (PMC12586762; doi:10.1111/iju.70207)
Supplement: Supplementary file 2 — Appendix S2: Landmark analysis of overall survival. [file IJU-32-1677-s002.docx]

Online Resource 2

Landmark analysis of overall survival

0

20

40

60

80

100

0

20

40

60

80

100

48

30

15

5

3

1

16

10

1

0

0

0

71

28

3

0

0

0

Immediate CN

Deferred CN

Without CN

Number at risk

Overall survival (%)

Time (months)

Without CN

Immediate CN

Deferred CN

P = 0.056
