# Supplementary material for: Role of Cytoreductive Nephrectomy in the Immune Checkpoint Inhibitor Era: A Multicenter Collaborative Study
Source: Int J Urol. 2025 Aug 19;32(11):1677–85. doi: 10.1111/iju.70207 (PMC12586762; doi:10.1111/iju.70207)
Supplement: Supplementary file 3 — Appendix S3: (a) OS of first‐line treatment following immediate CN (IO+IO or IO+TKI). (b) OS of first‐line treatment following deferred CN (IO‐IO or IO‐TKI). [file IJU-32-1677-s001.docx]

Online Resource 3a

0

20

40

60

80

100

0

20

40

60

80

100

Overall survival (%)

22

11

6

3

2

0

29

19

9

2

1

1

ICI-TKI

ICI-ICI

Number at risk


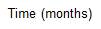


ICI-TKI

ICI-ICI

P = 0.17

Overall survivals of first-line treatment following immediate CN (ICI+ICI or ICI+TKI)

OS: overall survival

CN: cytoreductive nephrectomy

ICI: immune checkpoint inhibitor

TKI: tyrosine kinase inhibitor

Online Resource 3b

0

10

20

30

40

50

0

20

40

60

80

100

10

6

5

3

0

0

11

7

5

2

1

1

ICI-TKI

ICI-ICI

Number at risk


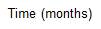


Overall survival (%)

ICI-TKI

ICI-ICI

P = 0.96

Overall survivals of first-line treatment following deferred CN (ICI-ICI or ICI-TKI)

OS: overall survival

CN: cytoreductive nephrectomy

ICI: immune checkpoint inhibitor

TKI: tyrosine kinase inhibitor
