# Supplementary material for: Role of Cytoreductive Nephrectomy in the Immune Checkpoint Inhibitor Era: A Multicenter Collaborative Study
Source: Int J Urol. 2025 Aug 19;32(11):1677–85. doi: 10.1111/iju.70207 (PMC12586762; doi:10.1111/iju.70207)
Supplement: Supplementary file 4 — Appendix S4: Uni‐ and multivariate Cox proportional hazards regression models for OS in the deferred CN group. [file IJU-32-1677-s004.docx]

Online Resource 4

**Uni- and multivariate Cox proportional hazards regression models for overall survival in the deferred CN group**

|  | **Univariate** | | **Multivariate** | |
| --- | --- | --- | --- | --- |
|  | HR (95%CI) | p-value | HR (95%CI) | p-value |
| **Female (yes)** | 5.66 (0.76-41.7) | 0.08 | 6.25 (0.67-57.7) | 0.11 |
| **Age ≥ 65 (yes)** | 1.2 (0.12-11.7) | 0.87 |  |  |
| **IMDC poor (yes)** | 1.3 (0.18-9.31) | 0.79 | 1.41 (0.13-15.1) | 0.77 |
| **Liver metastasis (yes)** | - | - |  |  |
| **N positive (yes)** | 0.77 (0.08-7.47) | 0.82 |  |  |
| **KPS ≤ 70 (yes)** | - | - |  |  |
| **Alb ≤ 4.0 mg/dl (yes)** | 1.06 (0.11-10.2) | 0.96 |  |  |
| **LDH ≥ 220 IU/L (yes)** | 1.62 (0.22-11.5) | 0.63 |  |  |
| **Clear-cell carcinoma (yes)** | - | - |  |  |
| **Sarcomatoid change (yes)** | 2.4 (0.24-24.01) | 0.456 | 2.04 (0.12-32.8) | 0.61 |
| **NLR ≥ 4.0 (yes)** | 0.69 (0.07-6.63) | 0.747 | 0.55 (0.04-6.51) | 0.63 |
| **CRP ≥ 4.0 mg/dl (yes)** | 0.36 (0.03-3.54) | 0.38 |  |  |

HR: Hazard ratio

CI: Confidence interval

IMDC: International Metastatic RCC Database Consortium

KPS: Karnofsky performance status

Alb: Albumin

LDH: Lactate dehydrogenase

NLR: Neutrophil-to-lymphocyte ratio

CRP: C-reactive protein
